# Targeted delivery of antibiotics to the infected pulmonary tissues using ROS-responsive nanoparticles

**DOI:** 10.1186/s12951-019-0537-4

**Published:** 2019-10-03

**Authors:** Yu Wang, Qian Yuan, Wei Feng, Wendan Pu, Jun Ding, Hongjun Zhang, Xiaoyu Li, Bo Yang, Qing Dai, Lin Cheng, Jinyu Wang, Fengjun Sun, Dinglin Zhang

**Affiliations:** 1 Department of Pharmacy, Southwest Hospital, Army Medical University (Third Military Medical University), Chongqing, 400038 China; 2 Department of Chemistry, College of Basic Medicine, Army Medical University (Third Military Medical University), Chongqing, 400038 China; 3 Department of Ultrasound, Southwest Hospital, Army Medical University (Third Military Medical University), Chongqing, 400038 China; 4 Department of Scientific Research Affairs, Army Medical University (Third Military Medical University), Chongqing, 400038 China; 5 Department of Pharmacy, Handan Branch of Chinese PLA 980 Hospital, Handan, 056000 Hebei China; 6 Department of Neurosurgery, Southwest Hospital, Army Medical University (Third Military Medical University), Chongqing, 400038 China

**Keywords:** ROS-responsive, Multifunctional nanomedicine, Targeted delivery, Antibacterial nanotherapy, Pulmonary infection

## Abstract

**Background:**

Immunocompromised individuals and those with lung dysfunction readily acquire pulmonary bacterial infections, which may cause serious diseases and carry a heavy economic burden. Maintaining adequate antibiotic concentrations in the infected tissues is necessary to eradicate resident bacteria. To specifically deliver therapeutics to the infected pulmonary tissues and enable controlled release of payloads at the infection site, a ROS-responsive material, i.e. 4-(hydroxymethyl) phenylboronic acid pinacol ester-modified α-cyclodextrin (Oxi-αCD), was employed to encapsulate moxifloxacin (MXF), generating ROS-responsive MXF-containing nanoparticles (MXF/Oxi-αCD NPs).

**Results:**

MXF/Oxi-αCD NPs were coated with DSPE-PEG and DSPE-PEG-folic acid, facilitating penetration of the sputum secreted by the infected lung and enabling the active targeting of macrophages in the inflammatory tissues. In vitro drug release experiments indicated that MXF release from Oxi-αCD NPs was accelerated in the presence of 0.5 mM H_2_O_2_. In vitro assay with *Pseudomonas aeruginosa* demonstrated that MXF/Oxi-αCD NPs exhibited higher antibacterial activity than MXF. In vitro cellular study also indicated that folic acid-modified MXF/Oxi-αCD NPs could be effectively internalized by bacteria-infected macrophages, thereby significantly eradicating resident bacteria in macrophages compared to non-targeted MXF/Oxi-αCD NPs. In a mouse model of pulmonary *P. aeruginosa* infection, folic acid-modified MXF/Oxi-αCD NPs showed better antibacterial efficacy than MXF and non-targeted MXF/Oxi-αCD NPs. Meanwhile, the survival time of mice was prolonged by treatment with targeting MXF/Oxi-αCD NPs.

**Conclusions:**

Our work provides a strategy to overcome the mucus barrier, control drug release, and improve the targeting capability of NPs for the treatment of pulmonary bacterial infections.

## Background

Immunocompromised individuals and those with lung dysfunction are susceptible to pulmonary bacterial infections, such as *Haemophilus species*, *Staphylococcus aureus*, and *Pseudomonas aeruginosa* [1]. After infection, numerous macrophages are recruited to the infection sites, and activated macrophages play a key role in the defense against invasive bacteria, viruses and fungi [2, 3]. Bacteria bound to the surface of macrophages are easily internalized into phagosomes and eventually digested [4]. However, some pathogens can escape host macrophage-mediated destruction through a TNF receptor-dependent mechanism [5]. Therefore, treatment with antibiotics (*e.g.*, moxifloxacin) is still an effective strategy to eradicate residual bacteria. Moxifloxacin (MXF), a broad-spectrum antibiotic, has enhanced activity against both gram-positive cocci (such as *Staphylococcus aureus, Streptococcus pneumonia, Streptococcus pyogenes,* and *Enterococcus faecalis*) and gram-negative pathogens (such as *E. coli* and *P. aeruginosa*) [6]. However, MXF is a hydrophilic drug that is easily cleared from the body within 24 h. To retain a sufficient concentration of MXF at the infection site, daily administration of MXF is necessary, which may result in dose-dependent side effects (such as hepatotoxicity) [7]. To solve this problem, nanoparticles (NPs) have been employed to encapsulate MXF to minimize the dosing frequency [8]. Nevertheless, the targeting capacity and controlled release behavior of MXF-loaded NPs need to be further optimized. To improve the targeting capability of NPs and eliminate intracellular pathogens, folic acid (FA)-modified NPs have been employed for targeted delivery of antibiotics to macrophages due to their overexpression of the folate receptors (FRs) [9, 10]. FRs are a family of glycoproteins (35–40 kDa), that can be divided into three isoforms: FR-α, FR-β and FR-γ [11]. FR-α is overexpressed on many types of cancer cells, including lung, breast, kidney, brain, endometrium, and colon cancers [11]. FR-α has also been detected in some normal tissues, such as kidney epithelial cells. Nevertheless, the kidney is protected from big size folate conjugates by their inability to be filtered through the glomerulus [12]. FR-β is overexpressed on activated macrophages. For example, Xia et al. demonstrated that only approximately 2% of F4/80^+^-resident peritoneal macrophages expressed FR-β after injection with sterile PBS, but 35% of F4/80^+^-resident peritoneal macrophages expressed FR-β after injection with live *P. aeruginosa* [13]. Both FR-α and FR-β exhibit strong affinity to FA. Therefore, FA-conjugated NPs and drugs have been widely used for imaging and targeted therapy of various diseases with infiltration of activated macrophage, such as inflammation, infection and tumor [14].

In addition to macrophages, neutrophils are also accumulated in the infection site due to the inflammatory response, resulting in the overproduction of reactive oxygen species (ROS), including hydroxyl radicals (·OH), hydrogen peroxide (H_2_O_2_), superoxide (O^2−^), and singlet oxygen (^1^O_2_) [15, 16]. ROS can inhibit the growth of microorganisms through oxidative damage of intracellular DNA [17, 18]. Unfortunately, ROS may induce multidrug resistance (MDR) by activating self-protective mechanisms in bacteria [19]. Furthermore, a high level of ROS can cause cytotoxicity by oxidation of DNA, lipids, proteins and other biomolecules, which is relevant to the pathogenesis of many diseases [20]. On the other hand, the rational utilization of ROS can realize “smart” release of payloads from carriers in pathological tissues with high ROS levels [21]. Thioether, selenium-containing polymers, poly(thioketal) and arylboronate can serve as ROS-responsive drug/gene nanocarriers because these materials can be oxidized or degraded under certain concentrations of H_2_O_2_ [22]. Among these materials, phenylboronic acid and polymers containing its esterification product exhibit excellent ROS-sensitivity under a biologically relevant range of H_2_O_2_ (0.5–1.0 mM) [23]. In our previous studies, we prepared phenylboronic ester-modified cyclodextrin and fabricated NPs, which can be rapidly decomposed even in the presence of 0.5 mM H_2_O_2_ [24, 25]. This finding suggested that our prepared materials can be used as drug carriers to achieve controlled release of payloads in pathological tissues with pathologically abnormal ROS levels [26].

**Fig. 1:**
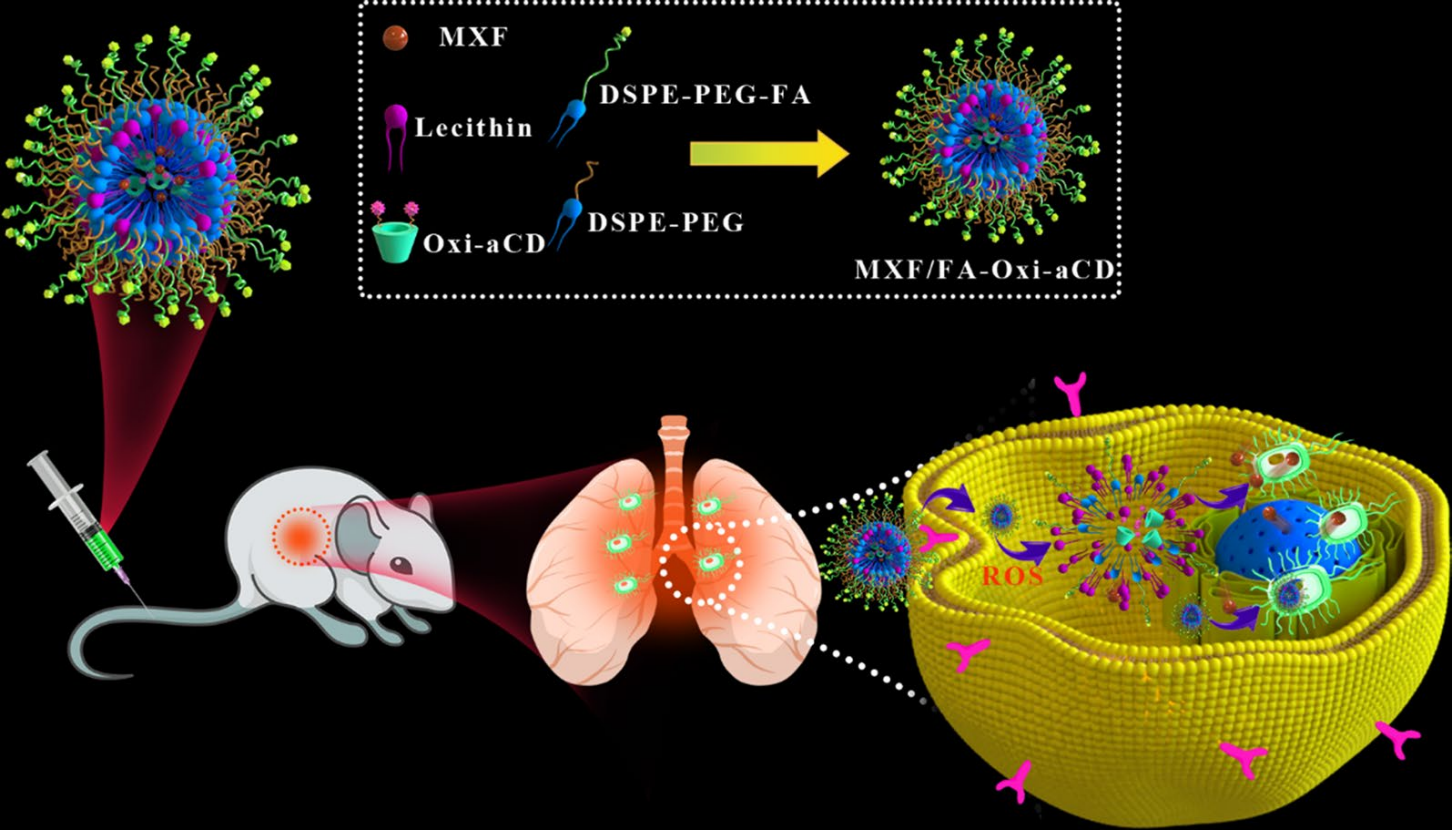
Schematic illustration of fabrication of ROS responsive MXF/FA-Oxi-αCD NPs and their application for targeted treatment of pulmonary *P. aeruginosa* infection

Herein a multifunctional nanotherapy was developed, to facilitate mucus penetration, efficiently deliver antibiotics to infected pulmonary tissues, and enable controlled release of payloads in the high-ROS microenvironment. To this end, 4-(hydroxymethyl) phenylboronic acid pinacol ester (HPAP)-modified cyclodextrin (Oxi-αCD) was used as a carrier to encapsulate MXF to prepare core–shell NPs (MXF/Oxi-αCD NPs) via a nanoprecipitation/self-assembly method (Fig. 1). The surface of MXF/Oxi-αCD NPs was coated with DSPE-PEG-FA and DSPE-PEG to achieve macrophage targeting and mucus penetration. The ROS-responsive NPs could smartly release their cargos at a high level of H_2_O_2_. In vitro antibacterial activity of nanoformulations was assessed with *P. aeruginosa.* MXF-loaded NPs exhibited enhanced antibacterial efficacy compared with free MXF. In vivo efficacy of MXF nanotherapies was evaluated in a murine model of pulmonary *P. aeruginosa* infection. FA-modified MXF/Oxi-αCD NPs can efficiently eradicate bacteria resident in pulmonary tissues and prolong the survival time of mice with pulmonary infection.

## Materials and methods

### Reagents

α-Cyclodextrin (α-CD) was purchased from Tokyo Chemical Industry Co., Ltd. (Tokyo, Japan). 1-(3-Dimethylaminopropyl)-3-ethylcarbodiimide hydrochloride (EDC. HCl), 4-(hydroxymethyl) phenylboronic acid pinacol ester (HPAP), 4-dimethylaminopyridine (DMAP), 1,1′-carbonyldiimidazole (CDI), poly(lactic-co-glycolic acid) (PLGA) and Pluronic F127 (a polyethylene oxide-polypropylene oxide-polyethylene oxide triblock copolymer, or PEO-PPO-PEO) were supplied by Sigma-Aldrich Co. (Shanghai, China). Lecithin (refined) was obtained from Alfa Aesar (Shanghai, China). 1,2-Distearoyl-sn-glycero-3-phosphoethanolamine-*N*-methoxy(polyethylene glycol)-2000 (DSPE-PEG_2000_) and folic acid-conjugated 1,2-distearoyl-sn-glycero-3-phosphoethanolamine-*N*-methoxy(polyethylene glycol)-3400 (DSPE-PEG_3400_-FA) were provided by Xi’an Ruixi Corporation (Xi’an, China). Cy5 free acid and Cy5-NHS ester were provided by Lumiprobe, LLC. (Hallandale Beach, FL, USA). LIVE/DEAD^®^ BacLight™ Bacterial Viability Kit (L7012) and SYTO 9 Green Fluorescent Nucleic Acid Stain were purchased from Thermo Fisher Scientific Inc. (Waltham, MA, USA). Dulbecco’s modified Eagle’s medium (DMEM) and fetal bovine serum were obtained from HyClone Inc. (Waltham, MA, USA). Streptomycin-penicillin solution was purchased from Solarbio Life Sciences Co., Ltd. (Beijing, China). 4′,6-Diamidino-2-phenylindole (DAPI) and a Hydrogen Peroxide Assay Kit (S0038) were provided by Beyotime Biotechnology Co., Ltd. (Shanghai, China).

### Cells and bacteria

The human lung epithelial cell line A549 and the mouse monocyte macrophage cell line RAW264.7 were obtained from the Cell Bank of the Chinese Academy of Sciences (Shanghai, China). Cells were incubated in DMEM supplemented with 10% fetal bovine serum, 100 µg/mL streptomycin, and 100 IU penicillin at 37 °C in a humidified atmosphere containing 5% CO_2_.

The following isolates were obtained from patients with pulmonary infections at the First Affiliated Hospital of the Army Medical University (Third Military Medical University): *P. aeruginosa* P304, P386, P582, P671, P723, P727 and P729; *K. pneumoniae* K253, K288 and K302; *E. coli* E282, E325, E611 and E640; and *S. aureus* S23 and S49. All the bacteria were incubated on Columbia blood agar plates at 37 °C. Then a single colony on the plate was picked and incubated in LB medium overnight at 37 °C. Subsequently, the concentration of bacterial solution was adjusted to 1.0 × 10^6^ colony forming units (CFU)/mL (Mc. turbidity) to determine the minimum inhibitory concentration (MIC) of MXF nanoformulations. The P727 isolate is a multidrug resistant strain, and therefore it was chosen to evaluate in vitro and in vivo antibacterial efficacy of MXF-loaded NPs in the lung infection with *P. aeruginosa*. A total of 1.0 × 10^6^ and 1.0 × 10^8^ CFU/mL of the P727 isolate was employed to infect A549/RAW264.7 cells and mice, respectively.

### Animals

Six-week-old female Kunming (KM) mice weighing 33 g were supplied from the experimental animal center of Army Medical University (Chongqing, China) and kept in an SPF-level sterile animal room. All animal experiments were performed in accordance with the guidelines approved by the ethics committee of Army Medical University (Chongqing, China).

### Synthesis of a ROS-responsive α-CD material (Oxi-αCD)

A ROS-responsive material was synthesized as previously reported [24]. In brief, HPAP (2.00 g, 8.5 mmol) was reacted with CDI (2.76 g, 17 mmol) in dry dichloromethane (DCM, 20 mL) to obtain CDI-activated HPAP (2.50 g). With DMAP (1.00 g, 8.1 mmol) as a catalyst, the Oxi-αCD material (0.735 g) was prepared by reacting CDI-activated HPAP (2.0 g, 6.1 mmol) with α-CD (0.33 g, 0.338 mmol) in DMSO (20 mL). The structure of the resulting material was confirmed by ^1^H NMR spectra.

### Synthesis of Cy5-labeled Oxi-αCD

Cy5 free acid (5.0 mg, 0.00963 mmol), DMAP (2.0 mg, 0.0164 mmol), and EDC. HCl (7.4 mg, 0.0385 mmol) were dissolved in 5.0 mL of DMF, into which 50.0 mg of α-CD (0.0514 mmol) was added and reacted at 25 °C for 2 days. After removal of the organic solvent, the residue was washed with acetone to obtain Cy5-conjugated α-CD. Using DMAP (150.0 mg 1.216 mmol) as a catalyst, Cy5-conjugated α-CD (50.0 mg) was reacted with CDI-activated HPAP (300.0 mg, 0.915 mmol) to obtain Cy5-labeled Oxi-αCD.

### Preparation and characterization of nanoparticles

A modified nanoprecipitation/self-assembly method was employed to prepare MXF/Oxi-αCD NPs [25]. Briefly, 6.0 mg of DSPE-PEG_2000_ and 4.0 mg of lecithin were dispersed in 400 µL of ethanol and 10.0 mL of deionized water, followed by preheating at 65 °C for 30 min. In parallel, 5.0 mg MXF and 50.0 mg Oxi-αCD were dissolved in 2.0 mL of methanol, and the obtained solution was added dropwise into the above preheated dispersion with vortexing for 3 min. After self-assembly for 2 h at 25 °C, MXF/Oxi-αCD NPs were harvested by centrifugation at 10,000 rpm (7620 *g*) for 10 min, washed with 5% F127 (10.0 mL) and resuspended in 0.2 mL of ultrapure water. Following similar procedures, Cy5-labeled Oxi-αCD NPs and blank Oxi-αCD NPs (without MXF) were prepared. In addition, FA-modified MXF/Oxi-αCD NPs (abbreviated as MXF/FA-Oxi-αCD NPs) were also fabricated with a similar method, in which 4.0 mg of DSPE-PEG_2000_ and 4.0 mg of DSPE-PEG_3400_-FA were used. Cy5-labeled and blank PLGA NPs were prepared through an emulsion solvent evaporation method. Briefly, in a 50 mL centrifuge tube, 5.0 mg of MXF or 1 mg of Cy5 dye, and 50.0 mg of PLGA, were dissolved in 0.7 mL of dichloromethane, then 7.0 mL of 1% PVA was added, followed by sonication for 2 min in an ice bath with a probe sonicator. The obtained emulsion was poured into 20.0 mL of 0.3% PVA and stirred for 2 h at 25 °C. PLGA NPs were harvested by centrifugation at 10,000 rpm (7620 *g*) for 10 min, washed three times with 10.0 mL of 5% F127 and resuspended in 0.2 mL of ultrapure water.

The size distribution and zeta-potential of NPs were measured by dynamic light scattering (DLS) analysis and laser Doppler electrophoresis, respectively (Malvern Zetasizer Nano ZS, Malvern, U.K.). The morphology of NPs was characterized by transmission electron microscopy (TEM) (JEM-1400, Japan).

### Drug loading and in vitro drug release study

To quantify the MXF content in NPs, 20 μL of fresh NPs suspension was lyophilized, weighed, and dissolved in 1.0 mL of methanol. The MXF concentration was measured by HPLC. Drug loading (DL) was calculated according to the following equation:

$$ {\text{DL }}\% \, = \,\left( {{\text{Amount of MXF in NPs}}/{\text{Weight of NPs}}} \right)\, \times \, 100\% . $$

To study the drug release behavior of NPs in vitro, 200 µL of suspensions containing newly prepared MXF/Oxi-αCD NPs or MXF/FA-Oxi-αCD NPs was separately added into dialysis tubing (MWCO: 3500 Da), which was immersed into 40.0 mL of PBS with or without 0.5 mM H_2_O_2_ at 37 °C. At predetermined time points, 4.0 mL of supernatant was withdrawn from the external medium and replaced with fresh medium. The concentration of MXF at each time point was determined by HPLC.

### Cytotoxicity evaluation by CCK-8 assay

RAW264.7 and A549 cells were seeded in DMEM at 1 × 10^4^ cells/well in 96-well plates for 24 h before treatment. Cells were coincubated with MXF and different NPs formulations at various concentrations for 24 h. Then 10 µL of CCK-8 solution was added into each well and cultured for another 1 h. The absorbance of cultures was detected at 450 nm using a Thermo Multiskan Spectrum spectrophotometer (Thermo Fisher Scientific Inc. MA, USA).

### Cell uptake

RAW264.7 and A549 cells were seeded at 1 × 10^5^ cells/well in 24-well plates and allowed to grow for 24 h. Then the cells were infected with the P727 isolate stained with SYTO 9 (at a ratio of 1:10) at 37 °C for 4 h. After infection, the cells were treated with free Cy5, Cy5/Oxi-αCD NPs, or Cy5/FA-Oxi-αCD NPs for 1 or 4 h. Cells treated with fresh culture medium served as a control. The treated cells were washed three times with 1.0 mL of PBS, fixed in 4% paraformaldehyde for 15 min, and rinsed three times with 1.0 mL of PBS. Cell nuclei were stained with DAPI for 10 min. After three times of washing with 1.0 mL of PBS, the cell uptake efficiency was detected by confocal laser scanning microscopy (CLSM) (Blue fluorescence, excitation at 405 nm and emission at 453 nm; Green fluorescence, excitation at 488 nm and emission at 561 nm; Red fluorescence, excitation at 633 nm and emission at 699 nm).

We also investigated the uptake behavior of RAW264.7 cells by flow cytometry. As described above, the infected cells were treated with cell culture medium (control group), Cy5, Cy5/Oxi-αCD NPs, or Cy5/FA-Oxi-αCD NPs for 1 h and then washed three times with 1.0 mL of PBS. The cells were harvested and detected by flow cytometry (Accuri C6, Becton, Dickinson and Company, USA).

### Determination of the minimum inhibitory concentration (MIC)

The MIC of MXF, blank NPs, MXF/Oxi-αCD NPs, and MXF/FA-Oxi-αCD NPs was separately determined using the agar dilution method [27]. Briefly, different concentrations of MXF in PBS were obtained from the stock solutions (initial concentration, 240 µg/mL) by serial dilution. Then, 1.0 mL of drug solution and 14.0 mL of Mueller–Hinton agar (MHA) were added into the cell culture dish and fully mixed. After solidification, all the bacterial suspensions (1 × 10^6^ CFU/mL) were inoculated with the antibacterial determiners. After incubation at 37 °C for 24 h, the inhibitory effects of these drugs were determined and calculated.

### Biofilm formation assays

P727 isolate cultures were adjusted to 1 × 10^6^ CFU/mL and inoculated in 96-well plates with LB broth in the presence of 1/4 × MIC of MXF or nanoformulations. After incubation at 37 °C for 24 h, the plates were rinsed three times with 1.0 mL of PBS and dried at 25 °C. Subsequently, the adherent cells were stained with 1% crystal violet (Sigma-Aldrich, USA) for 10 min and then rinsed three times with 1.0 mL of sterile water. After the plates were dried, the dye was dissolved in 30% acetic acid, and the absorbance of the solubilized dye was detected at 590 nm using a Thermo Multiskan Spectrum spectrophotometer (Thermo Fisher Scientific Inc. MA, USA). Each treatment was assayed in three wells per plate, and the experiments were repeated three times.

### Intracellular antibacterial activity assays

The antibacterial effects of MXF, blank NPs, MXF/Oxi-αCD NPs, and MXF/FA-Oxi-αCD NPs on intracellular infection were also tested. RAW264.7 cells were plated in 12-well plates with 2 × 10^5^ cells per well. In parallel, precultured bacteria were diluted with DMEM to 1 × 10^6^ CFU/mL. RAW264.7 cells were infected with diluted bacteria (at a ratio of 1:10) at 37 °C for 4 h. Cells treated with fresh culture medium were used as the control. Then, the supernatant was removed, and the residual cells were washed with 1.0 mL of gentamicin-containing PBS solution (100 µg/mL) three times to kill extracellular bacteria. The washed cells were then incubated with 1/4 × MIC of MXF, blank NPs, MXF/Oxi-αCD NPs or MXF/FA-Oxi-αCD NPs at 37 °C for 4 or 24 h. Subsequently, cells were harvested and lysed with Triton X-100 for 15 min. The cell suspensions were diluted 10-fold or 100-fold with saline and plated on agar plates at 37 °C for 24 h. The number of colonies was counted to assess the antibacterial effect of different formulations.

### Confocal laser scanning microscopy (CLSM)

Viability of the P727 isolate was tested using a LIVE/DEAD^®^ BacLight™ Bacterial Viability Kit (L7012) after treatment with various formulations. Briefly, the P727 isolates (1 × 10^6^ CFU/mL) were incubated with culture medium (control group), MXF, blank NPs, MXF/Oxi-αCD NPs, or MXF/FA-Oxi-αCD NPs at 1/4 × MIC of MXF for 4 h. Then a dye mixture (SYTO 9 dye and propidium iodide (PI) at a ratio of 1:1) was added to the bacterial suspension (at a ratio of 3 µL:1.0 mL). The bacterial suspensions were incubated for another 15 min in the dark at 25 °C and then observed using CLSM (Green fluorescence, excitation at 488 nm and emission at 522 nm; Red fluorescence, excitation at 561 nm and emission at 632 nm).

The interaction of Oxi-αCD NPs and bacteria was also recorded using CLSM. Briefly, the P727 isolate (1 × 10^6^ CFU/mL) was incubated with Cy5-labeled Oxi-αCD NPs for 2 h. The microscopic images were acquired by CLSM.

### Transmission electron microscopy (TEM)

The morphology of the P727 isolate after treatment with various formulations was observed by TEM (JEM-1400, Japan). After overnight culture, the bacterial suspension was diluted in saline to a concentration of 1 × 10^6^ CFU/mL. Then, bacteria were treated with 1/4 × MIC of MXF, blank NPs, MXF/Oxi-αCD NPs or MXF/FA-Oxi-αCD NPs for 4 h. An untreated bacterial suspension was used as the control. After removing the supernatant, the collected bacterial cells were fixed with 2.5% glutaraldehyde overnight, and the morphology of the bacterial cells was observed by TEM.

### Mucus penetration assay

The mucus penetration capability of Cy5-labeled Oxi-αCD NPs was determined using sputum from a patient with lung infection. Cy5-labeled PLGA NPs without PEG modification were used as the negative control. Cy5/Oxi-αCD NPs and Cy5/PLGA NPs were separately added to the sputum, and the distribution of these NPs was observed by CLSM at 0 and 3 h.

### Determination of intracellular and extracellular H_2_O_2_ generation in RAW 264.7 cells

To determine the intracellular and extracellular H_2_O_2_ concentrations, RAW 264.7 cells (2 × 10^5^ cells/well) were seeded in 12-well plates and cultured for 24 h. Then, the cells were infected with the P727 isolate for 4 h as previously described. After being washed with 1.0 mL PBS three times, the cells were incubated with various nanoformulations at 1/4 × MIC of MXF for 24 h. Cells without treatment were used as the control. The intracellular and extracellular concentrations of H_2_O_2_ were measured using a Hydrogen Peroxide Assay Kit according to the manufacturer’s protocol.

### Establishment of a murine model of pulmonary infection

A mouse model of pulmonary infection with the *P. aeruginosa* P727 isolate was established by a non-invasive tracheal intubation method. First, mice were administered an intraperitoneal injection of cyclophosphamide (Meilun, Liaoning Province, China) at 200 mg/kg body weight to induce immunosuppression 4 days prior to infection. Next, the immunosuppressed mice were anesthetized by an intraperitoneal injection of 1% pentobarbital (40 mg/kg, Sigma, USA). Then, 50 μL of P727 isolate solution (1 × 10^8^ CFU/mL) was injected into the lungs of the mice by orotracheal intubation under a transilluminating lamp. In contrast, the control group was treated with 50 μL of saline.

### In vivo biodistribution study

The bacteria-infected mice were randomly assigned to three groups that were treated with Cy5 solution, Cy5-labeled Oxi-αCD NPs, or Cy5-labeled FA-Oxi-αCD NPs via intravenous administration. The dose in each group was equivalent to 20 μg of Cy5 per mouse. At 2, 4, 6, 8, 12, and 24 h post injection, the mice were sacrificed. The main organs, including the heart, liver, spleen, lungs, and kidneys, were collected and rinsed with physiological saline. Then the Cy5 fluorescence intensity in organs was detected by a live animal imaging system (IVIS Spectrum, Perkin Elmer, USA) with a 640 nm excitation filter and a 700 nm emission filter.

### In vivo anti-infective activity

The pulmonary infection model in immunosuppressed mice was established as described above. After inoculation for 24 h, the infected mice were intravenously injected with saline, MXF, blank NPs, MXF/Oxi-αCD NPs, or MXF/FA-Oxi-αCD NPs at a dose of 5 mg/kg MXF once every 2 days post infection for a total of 2 treatments. Meanwhile, the control mice were treated with saline. The survival rate was determined by using the Kaplan–Meier method. After 6 days of treatment, the mice were sacrificed. The left lungs from the infected mice were harvested and fixed with 4% paraformaldehyde for H&E staining. If mice died during the experiments, the lung tissues was also collected for pathological analysis. The morphology of these organs was observed using an optical microscope (20×, Olympus, Japan). In addition, the right lung was weighed and homogenized under sterile conditions to determine *P. aeruginosa* bacteria counts. The homogenates were suspended in 1.0 mL of sterile saline, diluted quantitatively by serial dilution and then incubated on agar plates at 37 °C for 24 h. The number of colonies was counted 24 h later, and the results are presented as CFU/g lung weight.

### Statistical analysis

All data are presented as the mean ± standard deviation (SD) of at least three independent experiments. Statistical analysis was performed using one-way variance (ANOVA) for more than three groups and Student’s *t* test for two groups. Statistical significance was defined as *P < 0.05 and **P < 0.01.

## Results and discussion

### Synthesis and characterization of Oxi-αCD and Cy5-labeled Oxi-αCD

Our previous work reported that 4-(hydroxymethyl) phenylboronic acid pinacol ester (HPAP)-modified cyclodextrin possesses excellent biocompatibility and ROS responsiveness and could serve as a superior drug delivery vehicle [24–26, 28]. Herein, our previously reported Oxi-αCD material was employed to encapsulate MXF. The synthesis method of Oxi-αCD is illustrated in Additional file 1: Scheme S1 and its ^1^H NMR spectrum is shown in Additional file 1: Figure S1. To label the material with a fluorescent probe, Cy5 was conjugated onto α-CD with EDC.HCl and DMAP using as catalysts (Additional file 1: Scheme S2). As shown by the ^1^H NMR spectrum in Additional file 1: Figure S2, in addition to the proton signals of Oxi-αCD, the characteristic peaks of the methyl, double bond, and phenyl groups in Cy5 were clearly observed, indicating successful synthesis of Cy5-labeled Oxi-αCD.

### Fabrication and characterization of MXF/Oxi-αCD NPs

**Fig. 2:**
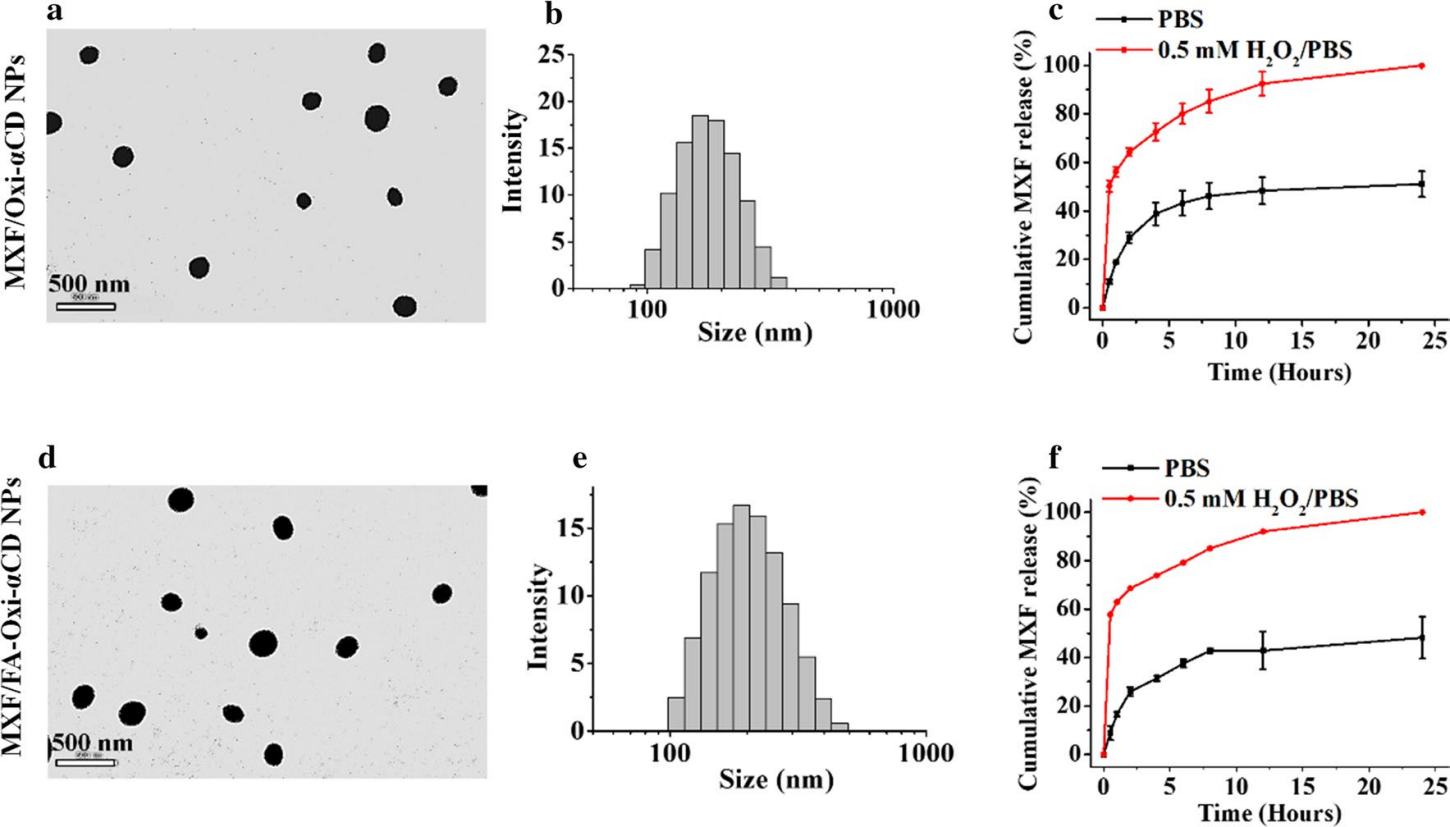
Morphology, size distribution, and drug release profiles of MXF/Oxi-αCD NPs and MXF/FA-Oxi-αCD NPs. TEM images indicated that MXF/Oxi-αCD NPs (**a**) and MXF/FA-Oxi-αCD NPs (**d**) displayed nearly spherical morphology. The size distribution of MXF/Oxi-αCD NPs (**b**) and MXF/FA-Oxi-αCD NPs (**e**). MXF encapsulated Oxi-αCD NPs (**c**) and FA-Oxi-αCD NPs (**f**) showed rapid release of MXF in 0.5 mM H_2_O_2_/PBS than PBS within 24 h. Data represent mean ± SD (n = 3)

MXF/Oxi-αCD NPs were fabricated via a nanoprecipitation self-assembly method (Fig. 1). The physicochemical characterization of NPs is summarized in Table 1. DLS measurements showed that blank NPs, MXF/Oxi-αCD NPs, and MXF/FA-Oxi-αCD NPs had a mean diameter of 203.8 ± 6.0, 266.2 ± 1.0, and 254.2 ± 9.5 nm, with a polydispersity index (PDI) of 0.184 ± 0.026, 0.162 ± 0.017 and 0.213 ± 0.008, respectively. All the NPs exhibited negative or low zeta-potential (from -32.4 to -37.4 mV) in 0.01 M PBS at pH 7.4. It is worth noting that Cy5-labeling did not significantly alter the size distribution, PDI, and zeta-potential of Oxi-αCD NPs. The morphology of MXF/Oxi-αCD NPs and MXF/FA-Oxi-αCD NPs was characterized by TEM. The TEM images confirmed that all the NPs were spherical and homogenous (Fig. 2a, d). DLS measurements also confirmed that these NPs were mainly distributed from 200 to 300 nm (Fig. 2b, e). HPLC quantification revealed that the MXF drug loading efficiency of Oxi-αCD NPs and FA-Oxi-αCD NPs was 5.78 ± 0.51% and 7.89 ± 0.45%, respectively (Table 1). The drug release behaviors of MXF/Oxi-αCD NPs and MXF/FA-Oxi-αCD NPs were also investigated. The release of MXF from MXF/Oxi-αCD NPs and MXF/FA-Oxi-αCD NPs was accelerated in 0.5 mM H_2_O_2_ (Fig. 2c, f), suggesting that controlled release of MXF from Oxi-αCD NPs occurred in the pathological tissues with high levels of H_2_O_2_. However, approximately 50% MXF was released from Oxi-αCD NPs in PBS within 24 h (Fig. 2c, f), perhaps because the hydrophilicity of MXF facilitated leakage from the NPs. Interestingly, a similar drug release rates were found for the FA-bearing and the non-targeted nanoformulations. In this study, the amount of FA in the total lipid was approximately 3% (weight ratio). Therefore, the introduction of FA did not significantly alter the physicochemical properties of the NPs, such as particles size, PDI, and drug loading (Table 1). FA-modification also did not change the hydrophobic properties of NPs. Therefore, FA-modified NPs had similar drug release behaviors as the non-targeted NPs.

**Table 1:** The physicochemical characterization of NPs

Nanoformulations	Particle size (nm)	Zeta-potential (mV)	PDI	Drug loading (%)
Blank NPs	203.8 ± 6.0	− 37.4 ± 0.7	0.184 ± 0.026	–
MXF/Oxi-αCD NPs	266.2 ± 1.0	− 22.4 ± 0.8	0.162 ± 0.017	5.78 ± 0.51
MXF/FA-Oxi-αCD NPs	254.2 ± 9.5	− 32.4 ± 0.4	0.213 ± 0.008	7.89 ± 0.45

Data represent mean ± SD (n = 3)

Macrophages infected with bacteria showed increased ROS generation [29]. To verify whether Oxi-αCD NPs can consume intracellular and extracellular hydrogen peroxide, the P727 isolate-infected RAW 264.7 cells were treated with blank PLGA NPs (non-ROS-responsive), blank Oxi-αCD NPs, MXF/Oxi-αCD NPs and MXF/FA-Oxi-αCD NPs. Compared to the control and blank PLGA NPs groups, the intracellular hydrogen peroxide concentration was decreased after infected cells were treated with ROS-responsive NPs (Additional file 1: Figure S3A), indicating these NPs can eliminate hydrogen peroxide. Also, the extracellular hydrogen peroxide concentration decreased significantly when infected cells were treated with ROS-responsive NPs compared to their counterparts (Additional file 1: Figure S3B). This may be attributed to the fact that the extracellular hydrogen peroxide concentration is far lower than the corresponding intracellular concentration, and this difference is obviously affected by carrier exhaustion. Furthermore, the cytotoxicity of MXF and the nanoformulations on RAW264.7 and A549 cells was evaluated. The viability of RAW264.7 and A549 cells incubated with MXF and nanoformulations showed no significant differences, indicating that Oxi-αCD NPs had no obvious cytotoxicity (Additional file 1: Figure S4).

### Cellular uptake

**Fig. 3:**
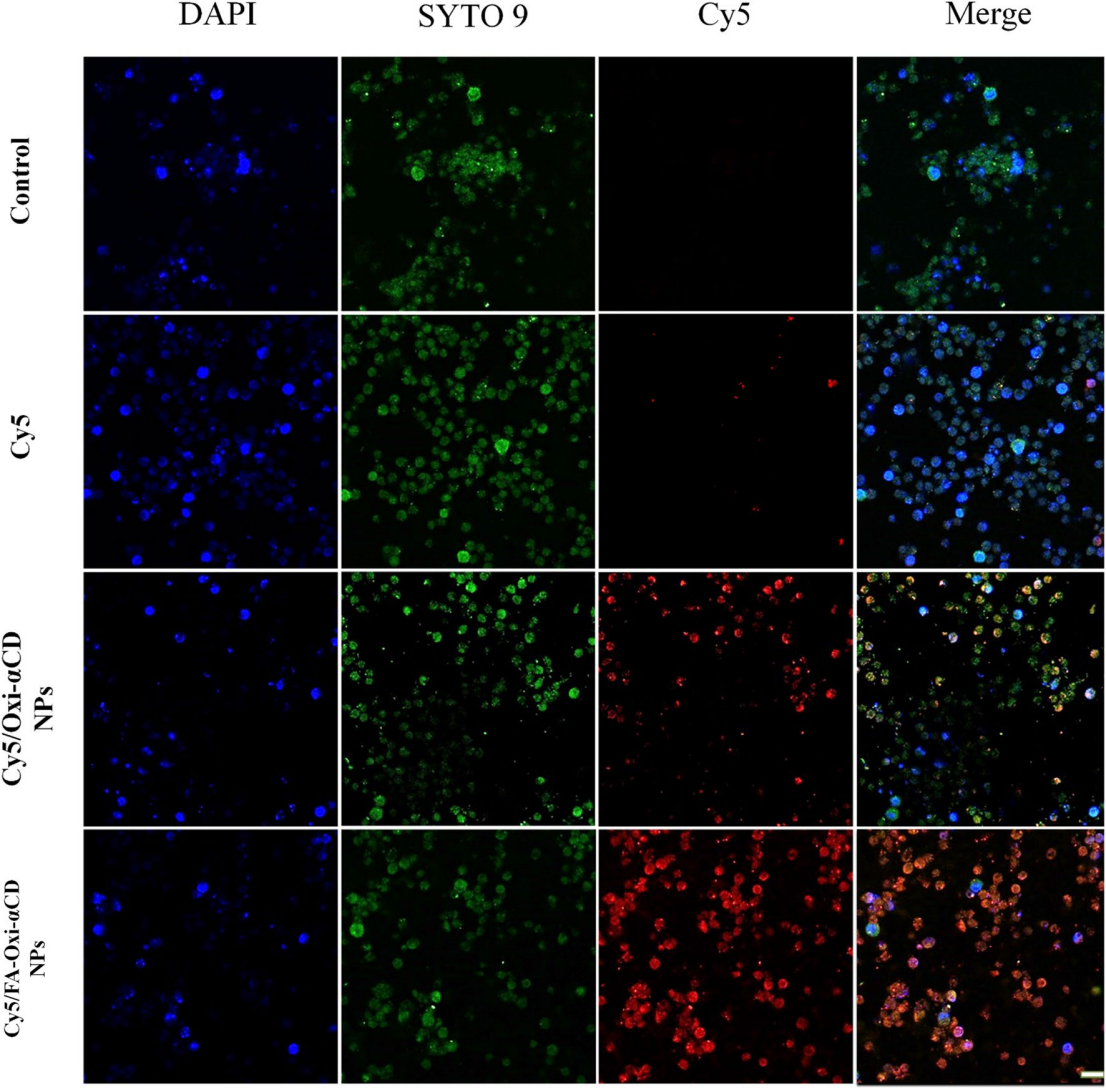
The CLSM images of P727 isolate-infected macrophages after separate treatment with culture medium (control), Cy5, Cy5/Oxi-αCD NPs and Cy5/FA-Oxi-αCD NPs for 4 h. RAW264.7 cells were infected with SYTO 9 stained P727 isolate at 37 °C for 4 h, then were incubated with Cy5 (0.05 μg/mL) and Cy5/Oxi-αCD NPs and Cy5/FA-Oxi-αCD NPs (containing 0.05 μg/mL Cy5) at 37 °C for 4 h. DAPI (blue), SYTO 9 (green), Cy5 (red). Scale bar represents 20 μm

Targeting delivery of antibiotics to macrophages is an effective strategy to eradicate cellular bacteria [9, 10]. It has been reported that FR is overexpressed on the surface of activated macrophages (i.e., bacteria and virus-infected macrophages) [9]. Modification of NPs with FA can enhance their targeting capacity to FR-overexpressing macrophages [11]. To target bacteria-infected macrophages, DSPE-PEG-FA was employed to coat Oxi-αCD NPs. To investigate whether FA-coating can enhance the cellular uptake of Oxi-αCD NPs by macrophages, the cellular uptake of free Cy5-NHS ester and Cy5-labeled Oxi-αCD NPs by bacteria-infected RAW264.7 (high FR expression) and A549 cells (low FR expression) was evaluated using CLSM. As shown in Additional file 1: Figure S5, after 1 h of incubation, compared with free Cy5, Cy5-labeled Oxi-αCD NPs and FA-Oxi-αCD NPs showed stronger red fluorescence signals inside RAW264.7 cells. This suggested that NPs could be efficiently internalized by macrophages. With prolonged incubation time to 4 h, the red fluorescence signals inside RAW264.7 cells were significantly increased, indicating that the uptake of NPs is time-dependent (Fig. 3). Remarkably, Cy5-labeled FA-Oxi-αCD NPs exhibited stronger fluorescence signals than non-targeted NPs at different time points (Additional file 1: Figure S5 and Fig. 3), implying that the internalization of FA-modified NPs was mediated by FR on the macrophage surface. Furthermore, the uptake of Cy5-labeled Oxi-αCD NPs and FA-Oxi-αCD NPs by A549 cells was also investigated (Additional file 1: Figure S6). A549 cells treated with Cy5/Oxi-αCD NPs and Cy5/FA-Oxi-αCD NPs had almost the same red fluorescence signals due to low FR expression on the surface.

Meanwhile, we quantified cell uptake efficiency using flow cytometry. Compared with Cy5, Cy5-labeled Oxi-αCD NPs and FA-Oxi-αCD NPs had higher uptake efficiencies (Additional file 1: Figure S7). Cy5-labeled FA-Oxi-αCD NPs achieved the highest uptake efficiency, indicating a consistent trend with the CLSM results. The abovementioned results suggested that FA-modified Oxi-αCD NPs could be efficiently internalized by bacteria-infected macrophages. Consequently, FA-modified Oxi-αCD NPs can serve as an effective drug delivery vehicles for targeted delivery of antibiotics to infected pulmonary tissues.

### In vitro antibacterial activities of the NPs

As described above, Oxi-αCD NPs can serve as an effective vehicles to deliver antibiotics to bacteria-infected macrophages. Therefore, we further investigated in vitro antibacterial activities of MXF-loaded Oxi-αCD NPs. The MIC value is an important parameter for evaluating the antimicrobial activity of drugs. Gram-negative and gram-positive bacteria isolated from patients with pulmonary infection were selected to test the MIC values of MXF and its nanoformulations. For all tested bacteria, the MIC of blank NPs was greater than 16 μg/mL (Table 2), implying that blank NPs have no antibacterial activity against the tested bacteria. However, MXF could significantly inhibit the growth of K288, E325, S23, P304, P723, and P729 bacteria, and the MIC of MXF for the above bacteria was less than 4 μg/mL. Compared with MXF, the MXF/Oxi-αCD NPs and MXF/FA-Oxi-αCD NPs had obviously decreased MIC values in MXF-sensitive bacteria. For example, the MIC of MXF for the S23 isolate was 0.25 μg/mL. The MIC of MXF/Oxi-αCD NPs and MXF/FA-Oxi-αCD NPs was decreased to 0.031 μg/mL. For the P723 isolate, the MIC of MXF/Oxi-αCD NPs and MXF/FA-Oxi-αCD NPs was 1 μg/mL, which is only one-quarter that of MXF. The results verified that MXF-loaded NPs had better antibacterial activity than free MXF. Similar MICs were observed for MXF/Oxi-αCD NPs and MXF/FA-Oxi-αCD NPs in the previously mentioned bacteria, indicating that FA modification did not alter the antibacterial activity of nanomedicines. Among these bacterial strains, *P. aeruginosa* is one of the most common pathogens related to bacterial infection in the lung. Due to the MDR of *P. aeruginosa*, the infected bacteria is difficult to completely eradicate from infection tissues [30]. According to our results (Table 2), P304, P386, P723, and P729 strains are sensitive to MXF. Therefore no significant difference in the antibacterial efficacy was found for MXF and MXF-loaded NPs. On the other hand, both MXF and MXF-loaded ROS-responsive NPs exhibited poor antibacterial effects on the P582 strain. Both the P671 and P727 strains showed resistant to MXF, but the P671 strain was sensitive to ciprofloxacin and levofloxacin, according to the results of the drug sensitivity tests in our hospital. In this study, we aimed to enhance the antibacterial efficacy of antibiotics and investigate the antibacterial mechanism of nanomedicines. Consequently, the P727 strain was selected as a model strain to further evaluate the antibacterial activity of MXF-loaded NPs.

**Table 2:** The MIC values of antimicrobial agents against clinically isolated bacteria

Strains	MIC (μg/mL)			
	Blank-NPs	MXF	MXF/Oxi-αCD NPs	MXF/FA-Oxi-αCD NPs
K253	> 16	16	8	8
K288	> 16	4	2	2
K302	> 16	> 16	> 16	> 16
E282	> 16	> 16	> 16	> 16
E325	> 16	2	0.5	0.5
E611	>16	> 16	16	16
E640	> 16	> 16	16	16
S23	> 16	0.25	< 0.03125	< 0.03125
S49	> 16	16	16	16
P304	> 16	4	2	2
P386	>16	8	4	4
P582	> 16	> 16	> 16	> 16
P671	> 16	16	4	4
P723	> 16	4	1	1
P727	> 16	16	8	8
P729	> 16	4	2	2

K, *K. pneumoniae*; E, *E. coli*; S, *S. aureus*; P, *P. aeruginosa*

**Fig. 4:**
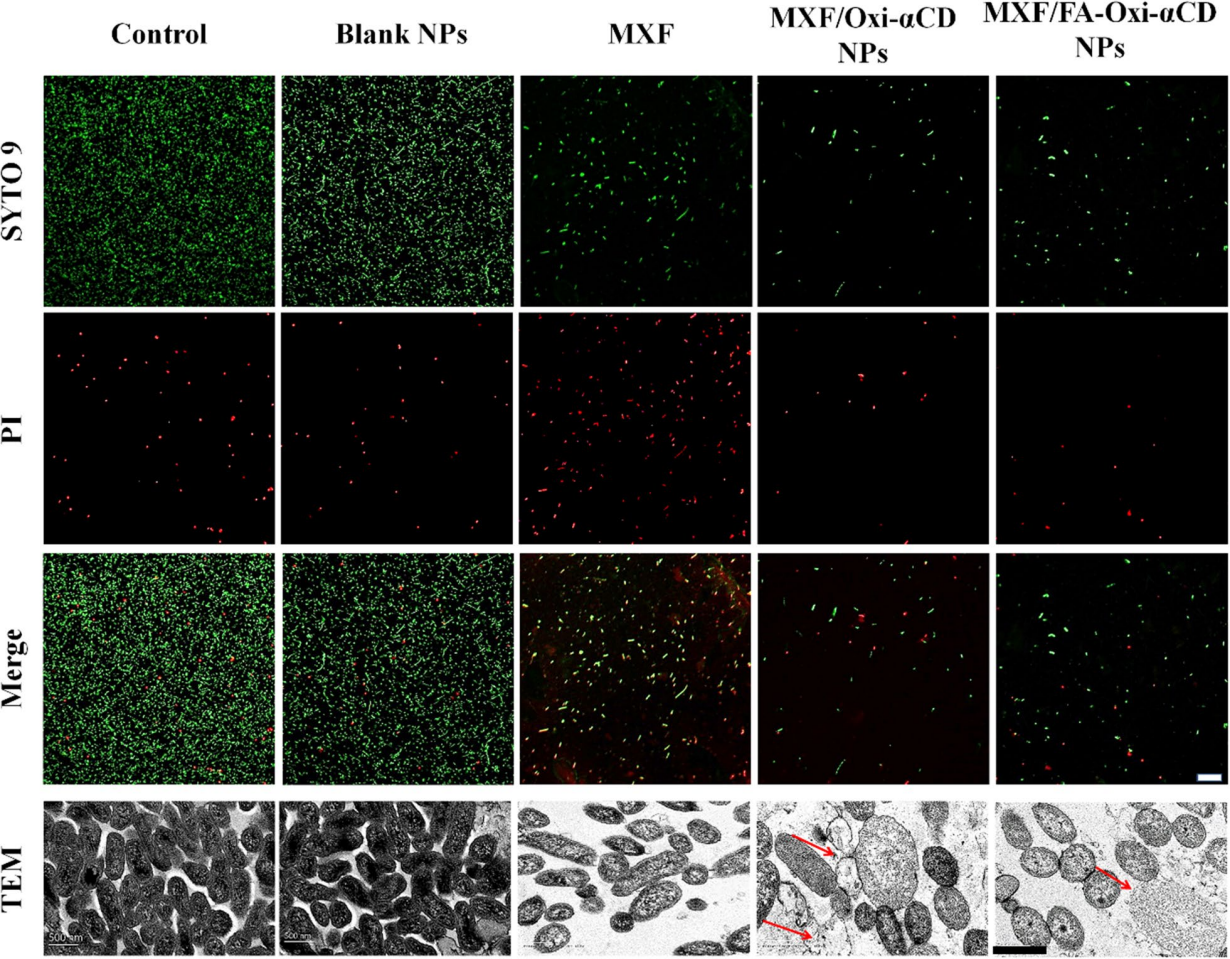
CLSM images of P727 isolate cells stained by SYTO 9 (green) and PI (red) as well as TEM images of P727 isolate cells. The P727 isolate cells were separately incubated with culture medium (negative control), blank NPs (without MXF-loaded Oxi-αCD NPs), MXF, MXF/Oxi-αCD NPs, and MXF/FA-Oxi-αCD NPs at 1/4 × MIC of MXF (4 μg/mL) for 4 h, then were stained by a dye mixture (SYTO 9 dye and PI at a ratio of 1:1) for 15 min for CLSM observation. As for TEM observation, the P727 isolate cells were fixed with 2.5% glutaraldehyde overnight. The red arrow indicates that P727 isolate cells had complete cellular surface disruption and the cellular cytoplasmic content of these cells underwent massive outflow, and only the cell membrane fragments remained. Scale bar represents 20 μm for CLSM images and 500 nm for TEM images

To further investigate the antibacterial activity of MXF and its nanoformulations, bacteria exposed to NPs were assayed using a LIVE/DEAD Bacterial Viability Kit and CLSM. To distinguish dead and live cells of the P727 isolate, live cells were stained with SYTO 9 (green), and dead cells were stained with PI (red). The stained P727 bacteria were observed by CLSM. As can be observed in Fig. 4, P727 bacteria treated with PBS or blank NPs showed a strong green fluorescence signal and weak red fluorescence, suggesting that blank NPs had no antibacterial effect. Compared to the control group, the MXF-treated group showed stronger red fluorescence and weaker green fluorescence, indicating that MXF can lead to P727 bacterial death. Interestingly, bacteria treated with MXF/Oxi-αCD NPs and MXF/FA-Oxi-αCD exhibited weaker green fluorescence signals than those treated with MXF, proving that these nanomedicines had stronger antibacterial activity than free drugs. Hong et al. investigated the antibacterial mechanism of NPs and proved that NPs could permeate the cell membrane of bacteria, thereby increasing their antibacterial efficiency [31]. MXF is a DNA topoisomerase inhibitor that inhibit bacterial growth by damaging cellular DNA [6]. The low MIC and high in vitro antibacterial activity suggested that a great amount of antibiotic could be delivered into bacterial cells by Oxi-αCD NPs. In addition, the antibacterial activity of MXF/Oxi-αCD NPs and MXF/FA-Oxi-αCD NPs against P727 bacteria was the same because FA modification did not increase the internalization of NPs by bacteria.

**Fig. 5:**
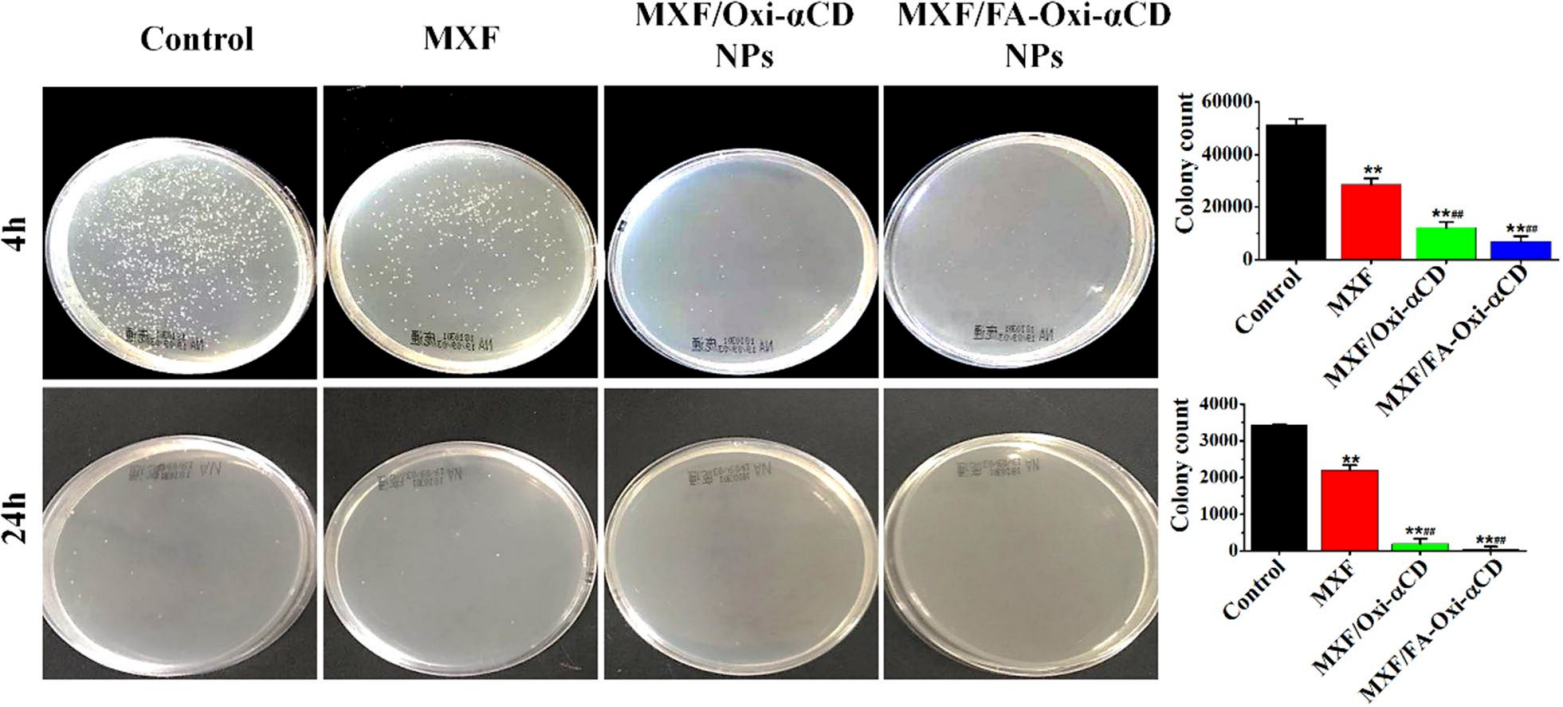
Digital images and quantification of colony counts of P727 isolate in activated macrophages after treatment with negative control (cells were treated with culture medium), MXF and its nanoformulations at 37 °C for 4 and 24 h at 1/4 × MIC of MXF (4 μg/mL). After treatment, cells were harvested and lysed with Triton X-100 for 15 min, then cell suspensions were diluted 10-fold or 100-fold with saline and plated on agar plates at 37 °C for 24 h. The number of colonies was counted to assess the antibacterial effect of different formulations. **, statistically different at *p *< 0.01 *vs* control; ^##^, statistically different at *p *< 0.01 *vs* MXF

As described previously, FA-modified Oxi-αCD NPs can be easily internalized by activated macrophages. To investigate whether our prepared nanomedicines can protect against cellular bacteria, P727 isolate-infected macrophages were employed to evaluate the antibacterial activities of MXF and its nanoformulations. P727 isolate-infected macrophages were treated with MXF, MXF/Oxi-αCD NPs or MXF/FA-Oxi-αCD NPs for 4 or 24 h, and the number of surviving bacteria in the infected macrophages was determined. Without any treatment, P727 bacteria showed rapid growth in macrophages (Fig. 5). The number of bacteria in the macrophages decreased with MXF treatment for 4 h, indicating that MXF exerted intracellular antibacterial activity. Compared with the MXF treatment group, the MXF/Oxi-αCD NPs and MXF/FA-Oxi-αCD NPs groups had fewer bacteria in the infected macrophages after 4 h of treatment, demonstrating that MXF/Oxi-αCD NPs and MXF/FA-Oxi-αCD NPs had better intracellular antibacterial activity than MXF. On the other hand, MXF/FA-Oxi-αCD NPs had slightly better antibacterial activity than MXF/Oxi-αCD NPs due to the increased internalization of FA-modified Oxi-αCD NPs by activated macrophages (Fig. 5). After 24 h of treatment, the number of intracellular bacteria in the control group was decreased due to the eradication of bacteria by macrophages [2, 3]. Interestingly, the number of bacteria in macrophages was significantly decreased by treatment with MXF-encapsulated NPs compared to MXF, and almost no intracellular bacteria were detected after MXF/FA-Oxi-αCD NPs treatment (Fig. 5). These results indicated that MXF-loaded Oxi-αCD NPs had greater intracellular antibacterial activity than MXF. The reason for this finding is that FA-modified NPs can easily adhere to FR on the surface of macrophages, leading to high internalization of MXF/FA-Oxi-αCD NPs by macrophages and enhanced antibacterial efficacy.

### Antibacterial mechanisms of MXF-loaded NPs

As previously described, MXF/Oxi-αCD NPs and MXF/FA-Oxi-αCD NPs had better antibacterial activity and lower MIC values than MXF. To clarify the antibacterial mechanism of MXF-loaded Oxi-αCD NPs, the morphology of bacteria after treatment with nanomedicines or control was observed by TEM. As shown in Fig. 4, cells in the control group had a normal, smooth cellular surface and a larger population. Compared with the control group, the group treated with 1/4 × MIC of MXF displayed partial cell surface disruption with slight leakage of cellular cytoplasmic content. Furthermore, bacterial cells treated with MXF/Oxi-αCD NPs or MXF/FA-Oxi-αCD NPs showed significant structural changes and complete cellular surface disruption. Meanwhile, the cellular cytoplasmic content of these cells underwent massive outflow, and only the cell membrane fragments remained (Fig. 4, red arrow). Nevertheless, the morphological structure of bacteria treated with MXF/Oxi-αCD NPs or MXF/FA-Oxi-αCD NPs showed no distinct difference. The cell membrane plays a key role in maintaining the normal function of bacteria, including providing a stable physiological environment, enabling the selective transport of substances, shielding water penetration and maintaining biological function. Some bacteria are difficult to kill due to complex cell envelopes with low permeability and extra defense mechanisms [32]. Herein, TEM images revealed that MXF/Oxi-αCD NPs and MXF/FA-Oxi-αCD NPs could disrupt the cell membrane of *P. aeruginosa* cells to inhibit bacterial growth.

Biofilms are widely associated with persistent bacterial infections with formidable resistance to conventional antiseptic drugs and local immune defense [33]. To further verify the antibacterial mechanism of MXF-loaded NPs, the biofilm formation of bacteria treated with NPs was assayed. As shown in Additional file 1: Figure S8, 1/4 × MIC of both MXF and nanoformulations suppressed biofilm formation by the P727 isolate. Interestingly, the inhibition of P727 biofilm formation by MXF/Oxi-αCD NPs and MXF/FA-Oxi-αCD NPs was much stronger than that by MXF and the control. *P. aeruginosa* can alter its outer membrane permeability to prevent antibiotic entry, resulting in antibiotic resistance [34]. These results indicated that MXF-loaded Oxi-αCD NPs may overcome the drug resistance of bacteria by suppressing bacterial biofilm formation.

As previously mentioned, MXF-loaded Oxi-αCD NPs showed better antibacterial activities than MXF through disrupting the cell membrane and suppressing bacterial biofilm formation. However, the underlying mechanism is still unclear. MXF is a DNA topoisomerase inhibitor, which exerts antibacterial activities by damaging cellular DNA. Therefore, we speculated that MXF-loaded Oxi-αCD NPs could effectively bind to bacteria and deliver more MXF molecules into cells. To test this hypothesis, the interaction of NPs and bacteria was directly observed by CLSM (Additional file 1: Figure S9). Compared with free Cy5, Cy5-labeled Oxi-αCD NPs and MXF/FA-Oxi-αCD NPs efficiently bound to bacteria. It has been reported that NPs can bind to bacteria through electrostatic, hydrophobic and van der Waals interactions [31]. Paunov et al. discovered that the antibacterial activity of copper oxide NPs was notably enhanced by boronic acid surface functionality because boronic acid can bind to the saccharide on the surface of bacterial cells [35]. Herein, the strong binding ability of ROS-responsive NPs to bacteria increased the uptake of NPs by bacteria, leading to more antibiotics entering bacterial cells and enhanced antibacterial activity.

### Evaluation of in vivo biodistribution

**Fig. 6:**
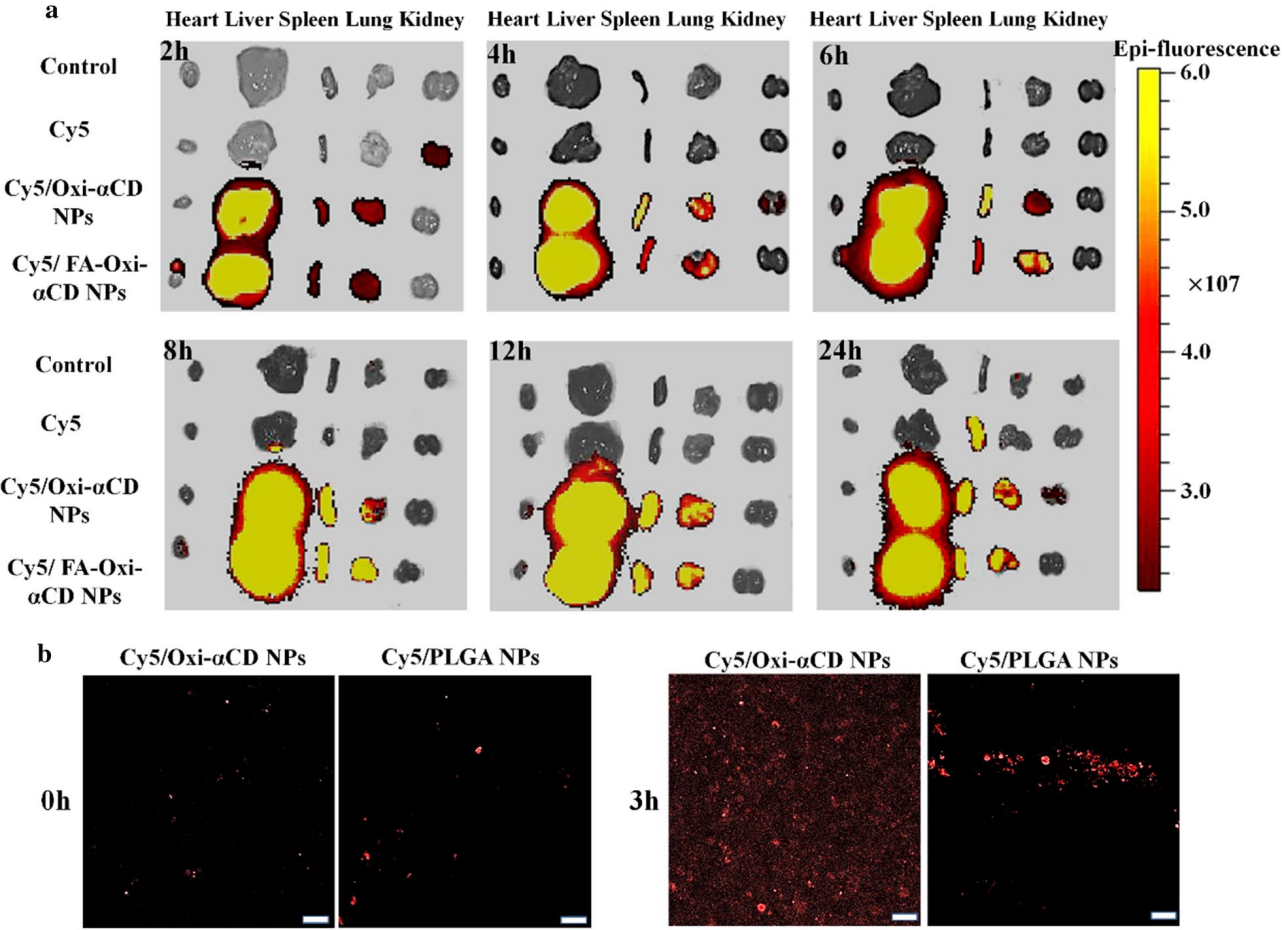
*Ex vivo* fluorescence images of excised organic tissues from mice with pulmonary P727 isolate infection after treatment with Cy5-NHS ester or Cy5-labeled nanoformulations (each mouse received 20 μg Cy5) at interval time point (**a**). The control was treated with saline. CLSM images of sputum dropwise with Cy5/Oxi-αCD NPs (PEGylated NPs) and Cy5/PLGA NPs (without PEG modification) with 0.05 μg/mL Cy5 at 0 and 3 h (**b**). Scale bar represents 20 μm

Pulmonary *P. aeruginosa* infection is usually accompanied by an inflammatory response, with abundant active macrophages accumulated at infection sites [2, 3, 36]. To investigate the targeting capability of NPs to the lung tissues, the biodistribution of Cy5-labeled NPs was analyzed in a mouse model of pulmonary infection using in vivo imaging assays. Ex vivo fluorescence images of excised tissues confirmed that mice injected with free Cy5 had weak fluorescence at different time points, suggesting that the free drug had low biodistribution in the lung tissues (Fig. 6a). Compared to the Cy5 group, the Cy5-labeled Oxi-αCD NPs group showed enhanced fluorescence in the lung tissues at 2 h post injection, and the fluorescence signals reached a maximum at 12 h post injection. After 24 h post injection, bright fluorescence signals were still observed in the lung tissues after treatment with Cy5-labeled Oxi-αCD NPs, indicating that NPs have longer retention times than the free drug. It is worth noting that mice injected with Cy5/FA-Oxi-αCD NPs displayed the strongest fluorescence intensity in the lung tissues at 4, 6, 8, 12 and 24 h post injection (Fig. 6a). These results proved that the targeting capability of Cy5/FA-Oxi-αCD NPs to infected lung tissues was increased by modification with FA, indicating that FA-modified Oxi-αCD NPs could serve as an effective platform to site-specifically deliver therapeutics to the infected lung tissues. However, strong fluorescence signals in the liver and spleen tissues were observed at different time points. Mikhail O Durymanov et al. summarized that NPs with diameters > 200 nm can be trapped by lung, spleen, and liver macrophages [37]. Enhanced uptake in the liver and spleen is attributed to macrophages, which play a crucial role in eliminating macromolecules and NPs circulating in the blood [38]. These factors resulted in high accumulation of Oxi-αCD NPs in liver and spleen tissues.

In some lung diseases, such as cystic fibrosis, lung function is compromised by the formation of purulent viscous mucus secretions, which benefit bacterial accumulation and survival [39]. The viscosity of mucus is increased because infected bacteria can stimulate the release of neutrophil chemoattractants from neutrophils and epithelial cells [40]. It is difficult for free drugs to penetrate the viscous mucus, leading to inadequate exposure of resident bacteria to antibiotics [41]. PEGylated NPs can efficiently penetrate the mucus due to the hydrophilic properties of PEG that can prevent the interaction between NPs and mucus (including DNA and other components), thereby improving the diffusion of NPs [42]. Therefore, the PEGylation strategy is widely used to treat mucus-heavy diseases such as pulmonary disease [39, 43], ocular disease [44], and vaginal disease [45]. DSPE-PEG-modified liposomes were also employed to treat mucus resident bacteria, such as *Helicobacter pylori* [46]. To penetrate mucus in the pulmonary tissues, we employed DSPE-PEG, a biocompatible material that has been extensively used as a drug/gene carrier to decorate NPs. The penetration ability of Cy5-labeled Oxi-αCD NPs in sputum was tested in vitro by CLSM via a layer-by-layer scanning model. At the beginning of the test, both Cy5/Oxi-αCD NPs and Cy5/PLGA NPs (no PEG coating) were assembled on the surface of sputum (Fig. 6b). After 3 h of incubation, Cy5/Oxi-αCD NPs penetrated through the sputum and dispersed in all directions. However, Cy5/PLGA NPs showed poor penetration and dispersion in the sputum (Fig. 6b). From these results, we demonstrated that PEGylated Oxi-αCD NPs could efficiently penetrate mucus and serve as a nanovehicles to deliver therapeutics to mucus-coated pulmonary tissues.

### In vivo anti-infective activity

**Fig. 7:**
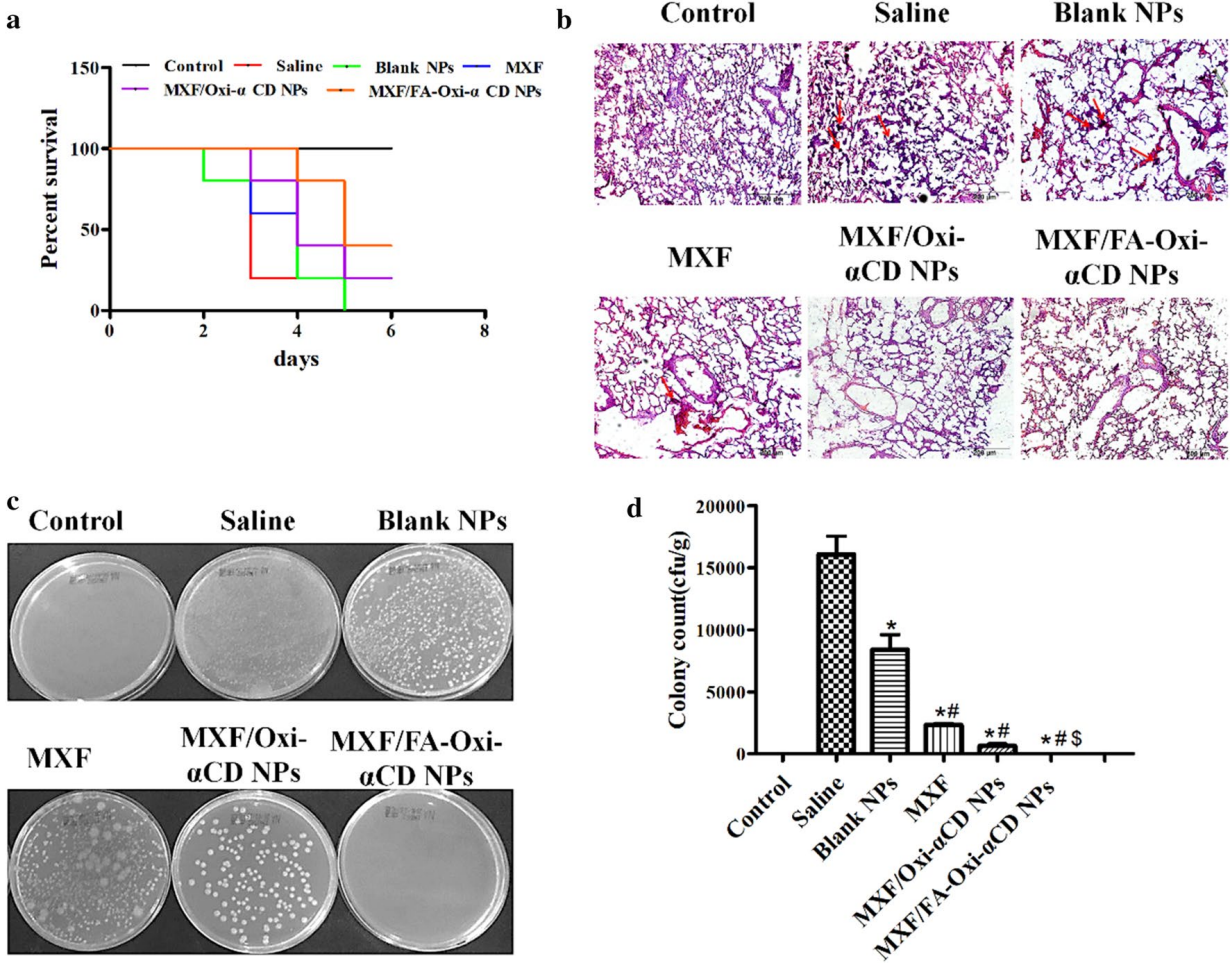
*In vivo* antibacterial efficacy of MXF and its nanoformulations in mice with pulmonary P727 isolate infection. Survival rate of bacteria-infected mice treated with saline, blank NPs (without MXF-loaded Oxi-αCD NPs), MXF, and MXF nanoformulations (each group included five mice, each mouse received two dose of free drug or MXF-loaded NPs with 5 mg/kg of MXF at interval 2 days, while mice in the blank NPs group received equal NPs with the drug-loaded NPs group) (**a**). H&E-stained sections of the lung tissues from normal and infected mice (**b**). The images and colony counts of P727 isolate of lung tissues (**c**, **d**). The red arrow indicates that P727 isolate colony was observed in the infected lung tissues. *statistical significance at *p *< 0.05 *vs* saline; ^#^statistical significance at *p* < 0.05 *vs* blank NPs; ^$^statistical significance at *p *< 0.05 *vs* MXF

These encouraging in vitro results on the antibacterial efficacy of MXF-encapsulated Oxi-αCD NPs prompted us to explore whether these nanomedicines are effective therapeutics for the treatment of pulmonary *P. aeruginosa* infection in vivo. To investigate whether MXF-loaded Oxi-αCD NPs could prolong the survival time of pulmonary *P. aeruginosa*-infected mice, a high concentration of bacteria (1 × 10^8^ CFU/mL) was injected into the lung tissues of immunosuppressed mice. The survival curve showed that infected mice treated with saline died within 48 h (Fig. 7a). Administration of MXF could prolong the survival time of mice to 6 days (20% survival rate), indicating that MXF has in vivo antibacterial efficacy. The survival rate of the mice treated with MXF/Oxi-αCD NPs or MXF alone was the same, while a 40% survival rate was obtained after treatment with MXF/FA-Oxi-αCD NPs. In vivo anti-infective experiments suggested that MXF/FA-Oxi-αCD NPs could increase the antibacterial activity of MXF.

After the experiment, all examined mice were sacrificed, and lung tissues were harvested to further investigate in vivo antibacterial efficacy of the MXF-loaded nanoformulations. The left lung tissues were collected for H&E staining and pathology analysis. Examination on H&E-stained sections of lung tissues revealed no significant pathological abnormalities or injuries in normal mice. However, a destroyed, incomplete alveolar wall structure, and many black bacteria colonies were observed in the lungs of mice infected with bacteria and treated with saline or blank NPs (Fig. 7b, red arrow). Compared with the saline or blank NPs group, the MXF, MXF/Oxi-αCD NPs and MXF/FA-Oxi-αCD NPs groups had relatively complete lung tissues structure and fewer bacterial colonies. In particular, almost no bacterial colonies could be found in the lungs after MXF/FA-Oxi-αCD NPs treatment (Fig. 7b). The right lung tissues were removed to quantitatively determine the bacteria count per weight (CFU/g). The colony count images showed fewer pulmonary bacteria in MXF-treated mice than those treated with saline or blank NPs. Moreover, mice treated with MXF/Oxi-αCD NPs had fewer pulmonary bacteria than MXF-treated mice. Remarkably, almost no bacteria were detected in the lungs of infected mice treated with MXF/FA-Oxi-αCD NPs (Fig. 7c, d). These results are consistent with the previous H&E results. Although MXF/FA-Oxi-αCD NPs could efficiently eliminate infected bacteria from the lung tissues, the survival time of these mice was only slightly prolonged compared to those in the free drug group. The reason may be that all the experimental mice were immunosuppressed and had a serious pulmonary bacterial infection and were too weak to survive for a long time.

All the results demonstrated that FA-modified ROS-responsive NPs can efficiently deliver MXF to infected lung tissues and enhance its antibacterial efficacy in a murine model of pulmonary *P. aeruginosa* infection. Therefore, MXF/FA-Oxi-αCD NPs can be further developed as a safe and efficacious nanomedicine for the targeted treatment of pulmonary diseases.

## Conclusion

Pulmonary bacterial infections seriously threaten the health of patients, especially immunocompromised patients and those with lung dysfunction. Targeted delivery of therapeutics to pulmonary tissues is a promising strategy to treat pulmonary bacterial infections. Herein, we developed a ROS-responsive nanoplatform to encapsulate MXF for the targeted treatment of pulmonary *P. aeruginosa* infection. MXF-loaded Oxi-αCD NPs can efficiently penetrate sputum due to the peripheral PEG coating. Furthermore, MXF can be rapidly released from NPs in the presence of H_2_O_2_. In vitro antibacterial assay demonstrated that MXF-loaded Oxi-αCD NPs have greater antibacterial efficacy than MXF against *P. aeruginosa*. In vivo biodistribution images showed that FA-modified Oxi-αCD NPs can be notably accumulated in infected lung tissues due to FA receptor overexpression on the surface of activated macrophages recruited to sites of inflammation. In a mouse model of pulmonary infection, MXF/FA-Oxi-αCD NPs can more efficiently eliminate bacteria from the lung tissues compared to MXF and MXF/Oxi-αCD NPs. The survival time of the mice was prolonged by treatment with MXF/Oxi-αCD NPs. Of note, the active targeting capability, mucus penetration ability, and controlled drug release of MXF/FA-Oxi-αCD NPs increased the drug concentration in pathological tissues and enhanced the antibacterial activity in vivo. In summary, FA-modified Oxi-αCD NPs can serve as an effective and safe drug delivery platform for the targeted treatment of pulmonary bacterial infection.

## Supplementary information

**Additional file 1.** Additional materials include the synthesis scheme of the materials and their ^1^HNMR spectra, intracellular and extracellular H_2_O_2_ concentration detection, cytotoxicity of the nanoformulations, CLSM images of the cell uptake of NPs, cell uptake efficiency determined by flow cytometry, biofilm formation, and CLSM images of bacteria interacted with NPs.
