# Supplementary material for: Targeted delivery of antibiotics to the infected pulmonary tissues using ROS-responsive nanoparticles
Source: J Nanobiotechnology. 2019 Oct 3;17:103. doi: 10.1186/s12951-019-0537-4 (PMC6777033; doi:10.1186/s12951-019-0537-4)
Supplement: Supplementary file 1 — Additional file 1. Additional materials include the synthesis scheme of the materials and their 1HNMR spectra, intracellular and extracellular H2O2 concentration detection, cytotoxicity of the nanoformulations, CLSM images of the cell uptake of NPs, cell uptake efficiency determined by flow cytometry, biofilm formation, and CLSM images of bacteria interacted with NPs. [file 12951_2019_537_MOESM1_ESM.docx]

**A ROS-responsive Moxifloxacin Nanomedicine for Targeted Treatment of Pulmonary *Pseudomonas Aeruginosa* Infection**

Yu Wang1, Qian Yuan1, Wei Feng1, Wendan Pu2, Jun Ding3, Hongjun Zhang4, Xiaoyu Li5, Bo Yang1, Qing Dai1, Lin Cheng1, Jinyu Wang6, Fengjun Sun1*, Dinglin Zhang2*.

1 Department of Pharmacy, Southwest Hospital, Army Medical University (Third Military Medical University), Chongqing 400038, China

2 Department of Chemistry, College of Basic Medicine, Army Medical University (Third Military Medical University), Chongqing 400038, China

3 Department of Ultrasound, Southwest Hospital, Army Medical University (Third Military Medical University), Chongqing 400038, China

4 Department of Scientific Research Affairs, Army Medical University (Third Military Medical University), Chongqing 400038, China

5 Department of Pharmacy, Handan Branch of Chinese PLA 980 Hospital, Handan, Hebei province 056000, China

6 Department of Neurosurgery, Southwest Hospital, Army Medical University (Third Military Medical University), Chongqing 400038, China

*Corresponding authors:

Fengjun Sun, PhD, Associate Prof.

Department of Pharmacy,

Southwest Hospital,

Army Medical University (Third Military Medical University), Chongqing 400038, China

E-mail: [fengj_sun@163.com](mailto:fengj_sun@163.com)

ORCID: 0000-0003-2679-8797

Dinglin Zhang, PhD, Associate Prof.

Department of Chemistry

College of Basic Medicine

Army Medical University, (Third Military Medical University), Chongqing 400038, China

E-mail: [zh18108@163.com](mailto:zh18108@163.com), [zh18108@tmmu.edu.cn](mailto:zh18108@tmmu.edu.cn)

ORCID: 0000-0003-4400-919X

Additional file 1: Scheme S1. Synthesis of Oxi-αCD material by conjugating HPAP onto α-CD.

Additional file 1: Figure S1. ^1^HNMR spectrum of Oxi-αCD material.

Additional file 1: Scheme S2. Synthesis of Cy5-labeled Oxi-αCD.

Additional file 1: Figure S2. ^1^HNMR spectrum of Cy5-labeled Oxi-αCD material.

A

B

Additional file 1: Figure S3. The intracellular (A) and extracellular (B) H_2_O_2_ concentration in the P727 isolate infected RAW264.7 cells treated with various nanoformulations at 1/4×MIC of MXF (4µg/mL) for 24 h. Cells treated with culture medium were used as the control group, the blank PLGA NPs and blank NPs group represent that cells were treated with PLGA NPs or Oxi-αCD NPs without MXF-loaded. *, statistically different at *p* < 0.05 *vs* control; #, statistically different at *p* < 0.05 *vs* Blank PLGA NPs. Data represents mean ± SD (n = 3)

A

B

Additional file 1: Figure S4. Cytotoxicity of various nanoformulations on RAW264.7 cells (A) and A549 cells (B) at different concentrations. The blank NPs group represnt that cells were treated with Oxi-αCD NPs without MXF loading. Data represents mean ± SD (n = 3)


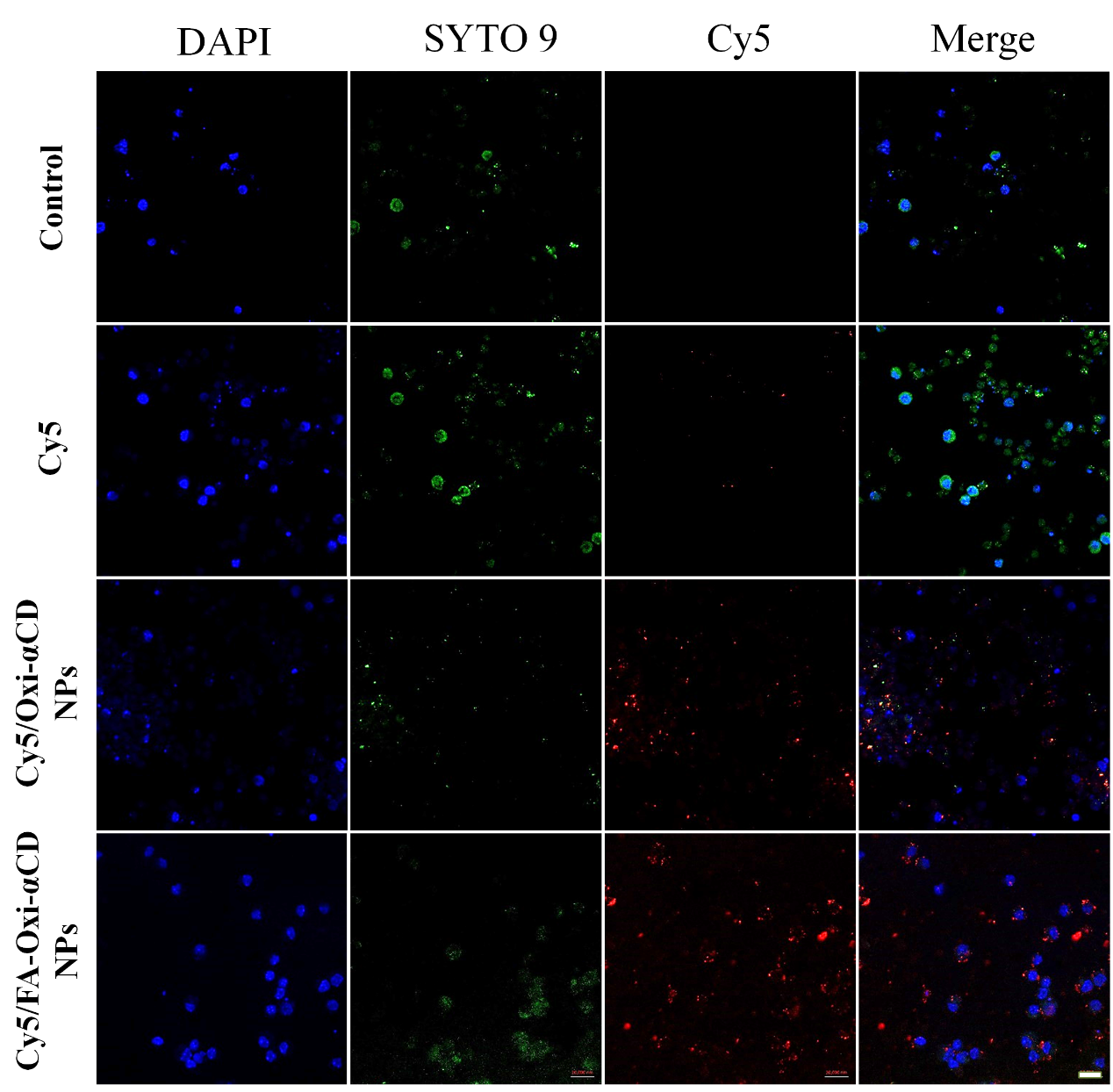


Additional file 1: Figure S5. The CLSM images of bacteria-infected RAW264.7 cells after treated with negative control (cells were treated with culture medium), Cy5, Cy5/Oxi-αCD NPs and Cy5/FA-Oxi-αCD NPs for 1 h. Raw264.7 cells were infected with SYTO 9 stained P727 isolate at 37°C for 4h, then were incubated with Cy5 (0.05 μg/mL) and Cy5/Oxi-αCD NPs and Cy5/FA-Oxi-αCD NPs (containing 0.05 μg/mL Cy5) at 37°C for 1 h. DAPI (blue), SYTO 9 (green), Cy5 (red). Scale bar represents 20 μm.


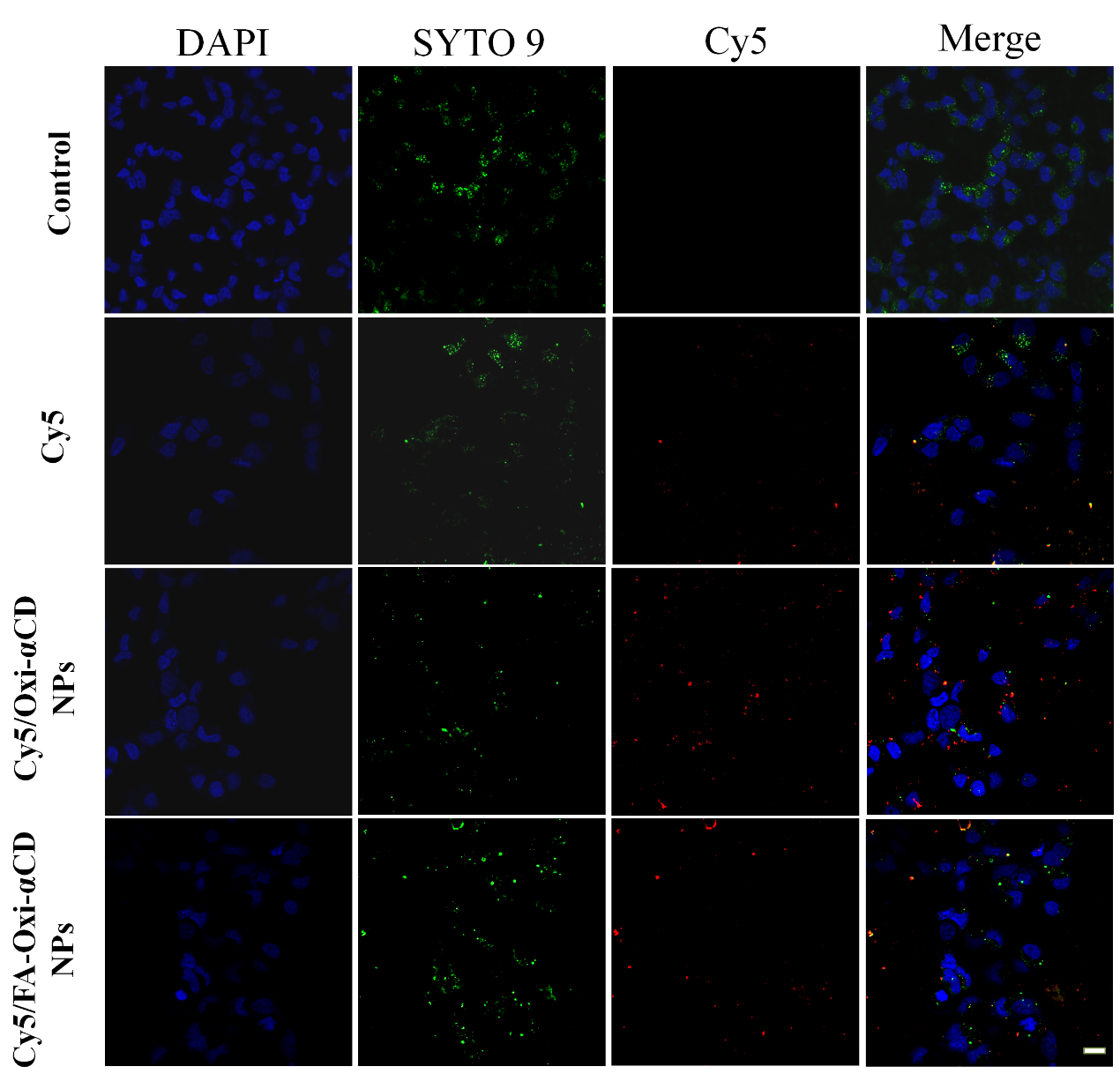


Additional file 1: Figure S6. The CLSM images of bacteria-infected A549 cells after treated with negative control (cells were treated with culture medium), Cy5, Cy5/Oxi-αCD NPs and Cy5/FA-Oxi-αCD NPs for 4 h. A549 cells were infected with SYTO 9 stained clinical isolate P727 at 37°C for 4h, then were incubated with Cy5 (0.05 μg/mL) and Cy5/Oxi-αCD NPs and Cy5/FA-Oxi-αCD NPs (containing 0.05 μg/mL Cy5) at 37°C for 4 h. DAPI (blue), SYTO 9 (green), Cy5 (red). Scale bar represents 20 μm.

Additional file 1: Figure S7. RAW264.7 cells uptake efficiency was determined using flow cytometry for 1 h. The P727 isolate infected RAW264.7 cells were treated with cell culture medium(control), Cy5(0.05 μg/mL), Cy5/Oxi-αCD NPs or Cy5/FA-Oxi-αCD NPs (both containing 0.05 μg/mL Cy5) at 37°C for 1 h. *, statistically different at *p* < 0.05 *vs* control; ^#^, statistically different at *p* < 0.05 *vs* Cy5; ^$^, statistically different at *p* < 0.05 *vs* Cy5/Oxi-αCD NPs. Data represents mean ± SD (n = 3)

Additional file 1: Figure S8. The effects on biofilm formation of P727 isolate treated with negative control (cells were treated with culture medium), MXF, MXF/Oxi-αCD NPs and MXF/FA-Oxi-αCD NPs at 1/4×MIC of MXF (4 μg/mL). **, statistically different at *p* < 0.01 *vs* control; ^#^, statistically different at *p* < 0.05 *vs* MXF. Data represents mean ± SD (n = 3)


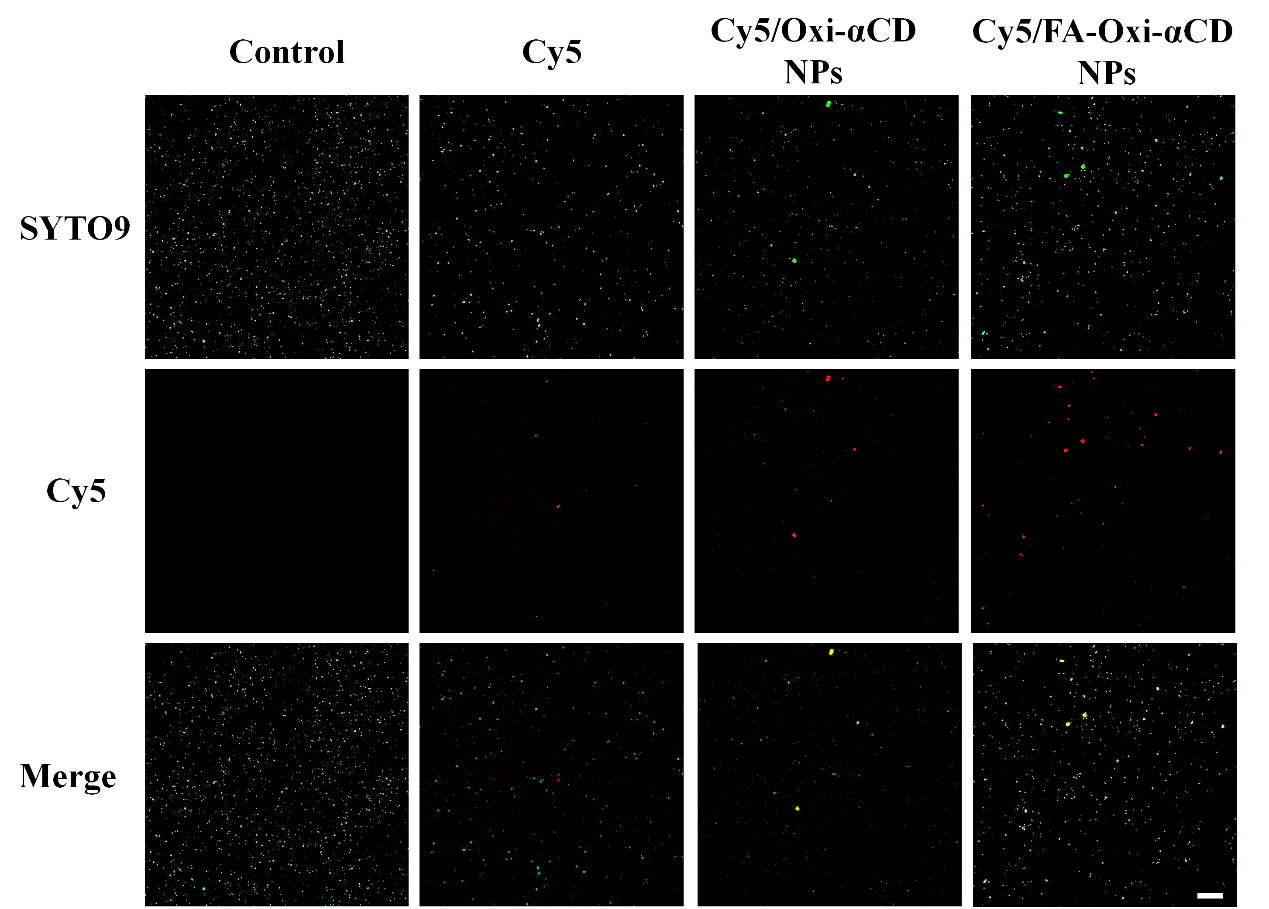


Additional file 1: Figure S9. The CLSM images of bacteria interacted with Cy5-NHS ester and Cy5-labeled NPs for 2h. Cells treated with culture medium were used as the control. The P727 isolate (106 CFU/mL) were incubated with Cy5-NHS ester (0.05 μg/mL), Cy5-labeled Oxi-αCD NPs and Cy5-labeled FA-Oxi-αCD NPs (both containing 0.05 μg/mL Cy5) at 37°C for 2 h. SYTO9 (green), Cy5 (red). Scale bar represents 20 μm.
